# A Novel Paradigm for Underwater Monitoring Using Mobile Sensor Networks

**DOI:** 10.3390/s20164615

**Published:** 2020-08-17

**Authors:** Anja Babić, Ivan Lončar, Barbara Arbanas, Goran Vasiljević, Tamara Petrović, Stjepan Bogdan, Nikola Mišković

**Affiliations:** 1 LABUST—Laboratory for Underwater Systems and Technologies, Faculty of Electrical Engineering and Computing, University of Zagreb, Unska 3, 10000 Zagreb, Croatia; ivan.loncar@fer.hr (I.L.); nikola.miskovic@fer.hr (N.M.); 2 LARICS—Laboratory for Robotics and Intelligent Control Systems, Faculty of Electrical Engineering and Computing, University of Zagreb, Unska 3, 10000 Zagreb, Croatia; barbara.arbanas@fer.hr (B.A.); goran.vasiljevic@fer.hr (G.V.); tamara.petrovic@fer.hr (T.P.); stjepan.bogdan@fer.hr (S.B.)

**Keywords:** marine robotics, swarm, multi-vehicle system, underwater acoustic sensor network, cooperative control, underwater monitoring, anomaly detection

## Abstract

This paper presents a novel autonomous environmental monitoring methodology based on collaboration and collective decision-making among robotic agents in a heterogeneous swarm developed within the project subCULTron, tested in a realistic marine environment. The swarm serves as an underwater mobile sensor network for exploration and monitoring of large areas. Different robotic units enable outlier and fault detection, verification of measurements and recognition of environmental anomalies, and relocation of the swarm throughout the environment. The motion capabilities of the robots and the reconfigurability of the swarm are exploited to collect data and verify suspected anomalies, or detect potential sensor faults among the swarm agents. The proposed methodology was tested in an experimental setup in the field in two marine testbeds: the Lagoon of Venice, Italy, and Biograd an Moru, Croatia. Achieved experimental results described in this paper validate and show the potential of the proposed approach.

## 1. Introduction

As the importance of studying the impact of climate change and anthropogenic influences on both a global and local scale grows, novel technologies find new application niches. The ecosystem of the Lagoon of Venice, Italy is critical in a scientific, cultural, and socio-economic context. It also stands out as an area with pronounced interplay between global climate change-related effects and a variety of unique site-specific phenomena [1,2].

Eutrophication and the effect of intense human activity on various types of sediment and soils present in the lagoon interact with increasingly intense summer heatwaves and abundant storms and rainfall during other times of the year, leading to a pronounced presence of phenomena such as degradation of zooplankton, abnormal proliferation of certain species of macroalgae such as *Ulva rigida*, and hypoxic and anoxic events in the form of rapid localised (usually overnight) drops in the concentration of oxygen in the water. These hypoxic and anoxic events are becoming more frequent and more spatially widespread, and have been tied to fish mortality and biomass reduction and shifts, making them a significant object of study [3,4,5,6].

Long-term environmental monitoring in the waters of the Venice Lagoon is a significant logistical challenge, demanding a great number of boats, personnel, and other resources. The Horizon 2020 Future and Emerging Technologies project subCULTron was conceptualised as a novel technological answer to these issues, in the form of an autonomous heterogeneous swarm of marine robots, which, when working together, create a topologically reconfigurable underwater acoustic sensor network with surface access points [7,8]. The subCULTron underwater swarm can also be seen as a part of the Internet of Underwater Things (IoUT), which is roughly defined as “a network of smart interconnected underwater objects” [9].

Monitoring an environment using an underwater swarm consists of two key parts. The first part is *exploration*—the ability of the swarm to autonomously move throughout the environment of interest, discover new areas, measure, and map them. The second part is *anomaly detection*—the ability of the swarm to detect interesting areas in the environment while exploring. Exploration usually includes a strategy on how to determine the next location to be visited and explored. Anomaly detection is by definition the problem of finding patterns in data that do not conform to expected behaviour. In order for a swarm to be truly autonomous, both of these parts need to be tackled autonomously. Finding an anomaly (outlier) in collected data can mean that either (i) the swarm is detecting changes in the environment that are interesting for analysis or (ii) that one or more sensors are faulty. An example of the former case is the occurrence of hypoxia/anoxia, where the anomalous area has low/no oxygen concentration measurements, in contrast to higher values present in the areas surrounding it.

Monitoring in underwater environments poses a set of unique challenges. While underwater, communication, which is the backbone of every swarm algorithm, is limited to low-bandwidth acoustic signals which are sensitive to signal interference, resulting in potential communication package loss. Since working with swarms means dealing with a large number of robotic units, it is also realistic to assume that these units are resource-constrained (limited processing power, storage, energy capacity), hence, any algorithms used should by necessity be of lower complexity. Since long-term monitoring involves a high probability of equipment damage and wear, none of the units should be a central point of failure, and there should be a method available for detection of faulty units. All of these factors lead to the conclusion that a distributed solution capable of fault-detection while operating online is necessary—and it is this solution that the subCULTron swarm aims to provide.

Several approaches to monitoring an underwater environment using a multi-robot system exist in the literature. One potential solution is using a heterogeneous system, such as those given in [10,11], where the authors use aerial, surface, and underwater robots, and deploy a system for gathering multi-domain data at coral reefs. The authors give a monitoring timeline with distinctive phases. A frequently seen approach, such as in [12,13], is using Lagrangian (current-following) drifters, capable of vertical movement.

In [14], the authors use an underwater sensor actuator network to detect extreme temperature gradients in a one-dimensional vertical setting. They propose an adaptive sampling algorithm based on binary search for the network to reliably detect a thermocline. The method is validated in a simple laboratory setting with mock-up sensor nodes and the authors do not consider potential sensor faults.

Systems similar to the one described in this paper have been developed for long-term underwater monitoring of coral reefs and fisheries [15] or oil exploration [16]. These approaches utilise fixed underwater sensors collecting data about the environment and Autonomous Underwater Vehicles (AUVs) acting as communication relays or data hubs tasked with the delivery of aggregated data to the surface. The system described in [15] employs two modes of communication (short-range optical and long-range acoustic) to better utilise the available energy on the sensing unit, thus increasing deployment longevity. The subCULTron system differs from the above-mentioned approaches by equipping its underwater sensor hubs with *z*-axis movement capabilities. This enables autonomous reconfiguration of the sensor network during mission execution either by utilising currents for drifting or using Autonomous Surface Vehicles (ASVs) as aids for relocation.

Exploration is a well-studied topic for ground and aerial vehicles, where most approaches consist of detecting the frontiers of the explored area and then estimating the utility of visiting each frontier point next. Utility balances information gain, travelling costs, and localisation ability for each point [17]. In an underwater setting, there are fewer approaches, focusing mostly on specific hardware properties [12,13,18] or exploration strategies [19]. Recently, in [20], the authors used information-theoretic exploration and sampling together with ocean models to derive an exploration strategy. An approach is given for a single-vehicle case, but a similar method could be adapted for a multi-robot setting as well.

An extensive overview of anomaly detection algorithms can be found in [21]. Only recently have truly distributed solutions started to emerge. These include consensus algorithms, such as trust-based consensus [22] and median consensus [23,24]. Agents agree on the value of interest (trust, median of all measurements) and outlier candidates are detected based on this value. Some approaches use movement capabilities of robots acting as sensor hubs for detection and tracing of anomalies [25] and neural network-based detection [26]. Similar to consensus, distributed Kalman filters can be used for state estimation and determination of outlier candidates through analysis of covariance matrices [27,28,29].

In this paper, exploration as a concept is only briefly discussed and the emphasis is instead placed on how the subCULTron swarm is designed to be continually moving through the environment and reaching new areas. In particular, strategies for deciding where to go next are not studied, as this topic requires extensive consideration and is a separate promising research direction.

The focus of the paper and the main contribution is a paradigm for distributed detection and verification of anomalies in the environment using a heterogeneous marine robot swarm. As opposed to other anomaly detection algorithms that operate on a given static data set, the motion capabilities of robot units and the reconfigurability of the swarm are exploited to collect additional data and verify the nature of the initially-obtained anomalies—or detect potential sensor fault among the swarm agents. As an important real-world application of the swarm is detecting and monitoring the anoxia phenomenon in the Lagoon of Venice, this proposed paradigm was tested in the field using an analogous proof-of-concept experimental scenario. Even though the approach is described as implemented and used by the subCULTron system, concepts can be transferred to other robotic systems as well.

The paper is structured as follows. Section 2, Materials and Methods, contains a description of the subCULTron robotic system Section 2.1 and an outline of the proposed paradigm and resulting experimental scenario Section 2.2. The experimental setup and results are given and discussed in Section 3. The final section contains a conclusion and directions for future work.

## 2. Materials and Methods

### 2.1. Subcultron System Description

**Figure 1:**
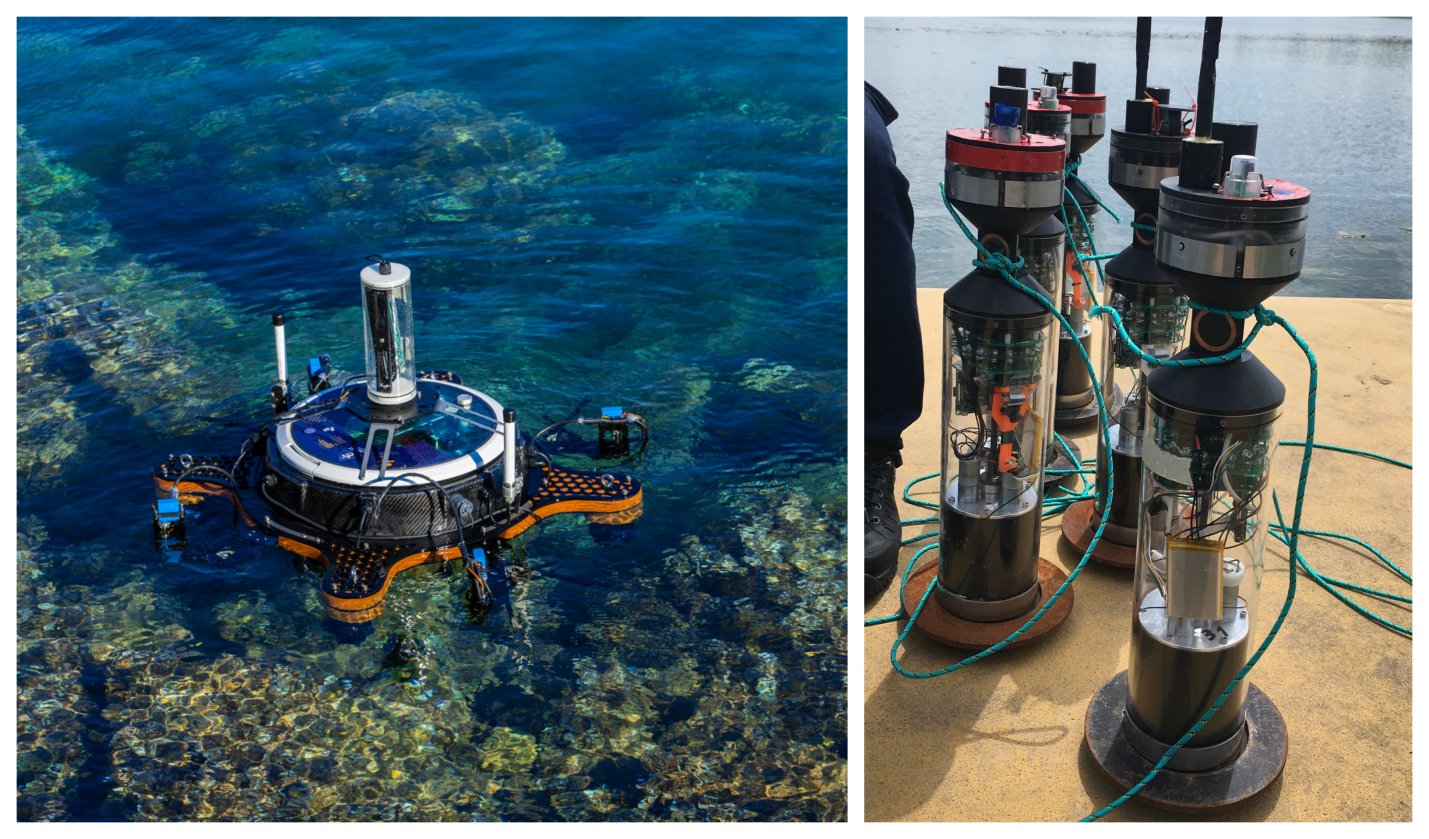
aPad (**left**) and aMussel (**right**) robots.

The subCULTron multi-agent system [30] was envisioned as an artificial marine ecosystem, aiming to reduce the need for boats and personnel manually deploying and collecting measuring instruments at sea by automating the deployment, reallocation, and collection of monitoring devices, as well as data acquisition. Besides its autonomy, this system has another significant advantage inherent to its large structure, which is the ability to measure environmental factors from multiple locations simultaneously and to reconfigure its topology dynamically. The system is comprised of three layers: five Autonomous Surface Vehicles (ASV) called aPads (artificial lily pads) constitute the top layer, a small swarm of aFish (artificial fish) represent the middle layer, and more than 100 underwater sensor nodes called aMussels (artificial mussels) act as the bottom layer. The monitoring paradigm proposed in this paper makes use of the aPad and aMussel agent types. A detailed description of the functionalities and agent interactions featuring the aMussel and aPad robots (Figure 1), as well as the subCULTron system as a whole, is presented in [31]. Here, an overview is given to provide necessary context.

#### 2.1.1. AMussel

**Figure 2:**
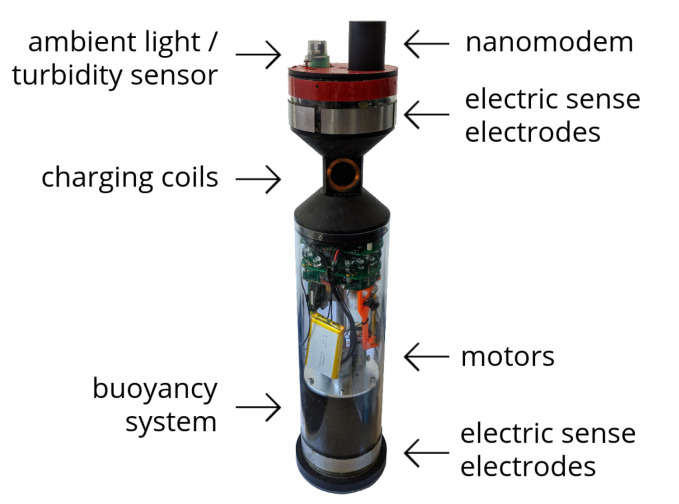
The aMussel robot—sensors, actuators and communication devices.

The aMussel agents serve as low cost underwater perception hubs, working together to establish a sensor network for distributed environmental monitoring and long-term data collection. As the primary measurement devices, they house a variety of sensors capable of monitoring relevant environmental factors. The design of the aMussel is represented in Figure 2. For the application described in this paper, the oxygen concentration, pressure, temperature, and turbidity sensors were installed, but any other compatible sensors can easily be added due to the robot’s modular design. For long-range communication, aMussels rely on miniature acoustic modems called nanomodems while submerged, and have Bluetooth, GSM, and WiFi capabilities when on the surface.

**Figure 3:**
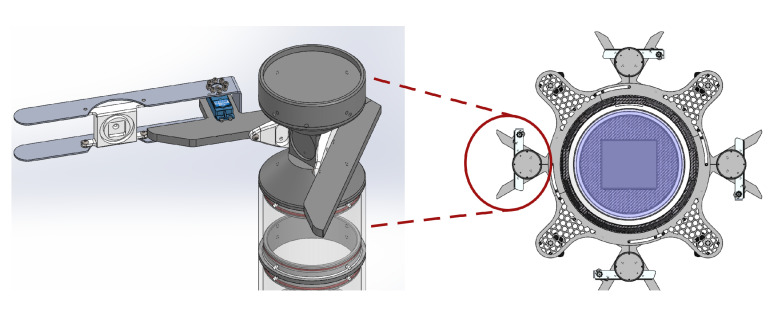
aPad docking mechanism design. Details show an aMussel top cap being grasped.

The aMussel was developed with long-term autonomy in mind, which is imperative for successful monitoring of environmental processes with slow dynamics. Its main electronic board, called the MU (Measurement Unit) board, is capable of deep hibernation, which drastically reduces energy expenditure. The rest of the modules connected to the central board can be independently powered off from the central unit, providing additional energy saving. During its operation, an aMussel may switch between modes of sleep and active perception and communication, depending on the application scenario. Each aMussel unit also carries a Raspberry Pi board with a custom-made adapter board, powered on in short intervals to provide additional processing power and data storage. To further prolong the operation of aMussel agents, a unique docking mechanism with inductive coils was devised, using which an aPad can grasp the aMussel and charge it (Figure 3). Recent related work [32] regards the analysis of long-term autonomy of the system, mainly from the standpoint of energy exchange between aPad and aMussel robots. The paper contains an examination of data acquired during efforts to model subCULTron swarm agent batteries as well as validate the long-term potential of the developed system. Of particular interest, is a single aMussel battery discharging with the MU board turned on but idle, with approximately 16 h needed for full discharge, and the aMussel battery discharging with the MU board in sleep mode, waking up once every hour for about 10 s in order to record measurements from all sensors, where 10 days were needed for full discharge. While taking into account the batteries’ ageing and ability to hold charge [33], switching to and actively using the aMussel’s identical secondary battery after its primary has been depleted effectively doubles this total deployment time. Since the energy exchange abilities of the agents greatly impact the longevity of the aMussel portion of the swarm, several cases of the aMussels recharging their batteries were also recorded. Here, of particular interest was the aMussel primary battery charging with the MU board turned on but idle (approximately 9.5 h needed for full charge), the aMussel primary battery charging with sleep, waking up briefly in regular intervals to record sensor data (6.5 h), and the aMussel secondary battery charging with the same sleep behaviour, with the MU powered by the primary battery (8 h). For comparison, battery data of an aPad running its on-board computer and Kinect camera and activating all of its thrusters for 3 s every 30 s was recorded, leading to a result of complete discharge after 27 h.

Since aMussels are mainly concerned with collecting data while resting at the seabed, their design involves limited movement capability, with a buoyancy system which allows them to sink to the seafloor, float to the surface, or hover at a certain depth being their sole actuator. They rely on assistance from the aPads for any more complex movement: when surfaced, the aMussel can be grasped and transported by an aPad, with the same docking mechanism used for energy transfer (Figure 3).

The aMussel user software runs on the MU Board with a Cypress PSoC4 (Programmable System-on-Chip) microcontroller running FreeRTOS and Embedded C software. PSoC programming tools allow easy-to-use graphical software redesign when changes in hardware occur. A high-level Application Programming Interface (API) written in C++ enables secure and reliable access to all sensors and actuators and keeps implementation details of device management hidden away from algorithm designers.

#### 2.1.2. APad

The aPad, pictured left in Figure 1, is an overactuated Autonomous Surface Vehicle (ASV). The primary role of the aPad in the swarm is to aid and assist underwater units, primarily by supplying energy and movement capability. They also serve as a link to any potential human observer and operator, as they take part in both the underwater and surface communication network. Below the surface, aPads communicate using the same nanomodem devices that are present on the aMussel units. For surface communication purposes, they have a mesh-capable wireless router which provides both WiFi access points for aMussels and surface stations and a mesh network for the aPads themselves.

The platform has four thrusters in an X-shaped configuration which make it overactuated and omnidirectional. Each aPad has four mechanical docking stations with inductive transmitter coils, and can charge up to four aMussels at a time. To enable autonomous grasping of surfaced aMussels, besides the mechanical docking station design, a visual servoing system was devised comprising a Kinect sensor and a specially designed pan mechanism [34].

Another vital role of the aPads is one of anchors in the process of underwater localisation [31]. In underwater sensor localisation, anchors positioned at known positions provide the source of information necessary for the localisation of unlocalised underwater nodes. Underwater nodes then collect measurements to gather information about the ranges between them and anchors. Then, using these measurements and simple algorithms such as multilateration, the unknown node positions can be calculated. For their own localisation and positioning, each aPad has an IMU and a GPS module. With its localisation and navigation capabilities, the aPad can return to a home position and have its batteries recharged while deployed thanks to a waterproof charging jack on its hull.

The aPad software runs on an Intel NUC mini PC, its main on-board computer. Similarly to the aMussel, the code is structured hierarchically and provides end-users with a simple API written in the Python programming language, enabling high-level mission planning and execution. Lower-level controllers, thruster allocation, sensor drivers and utilities, and navigation filters are implemented in C++ and Python and organised in packages under the Robot Operating System (ROS) paradigm.

### 2.2. Formal System Description

This section describes the proposed paradigm for underwater monitoring. The goal of the system is to find and confirm anomalies in a certain area of the seabed. The first step is for the aPads to deploy a group of aMussels, called the exploration group, to different predefined locations. After deployment, this group sinks to the seabed and begins an acoustic exchange of measurements. If one aMussel detects anomalous measurements, it becomes an outlier candidate. In order to validate the outlier candidate, a new group of aMussels, called the verification group, is deployed by aPads to its position. The verification group of aMussels exchanges measurements with the outlier candidate and either confirms or disproves that the area to which they have been deployed is anomalous. The final step includes the relocation of the outlier candidate, either to a home position for repair if the outlier is disproved, or to a new location to explore if the anomaly is confirmed and hence the outlier agent is “trusted”.

Due to the low cost and high number of the aMussel units, it is justified to use a certain number of them for verification of potential anomalies, as well as for future monitoring of a confirmed anomaly. In addition, it is possible to dynamically reallocate aMussels from the verification group to the exploration group in cases where there is a need to explore larger areas.

The subCULTron swarm used in in this paper consists of two types of robotic agents: aPads $P=\{P_{1},P_{2},P_{3},\ldots .,P_{n}\}$ and aMussels $M=\{M_{1},M_{2},M_{3},\ldots .M_{m}\}$, where *n* is the number of aPads used and *m* is the number of aMussels used.

The state of each aPad $P_{i}$ in time step *k* can be described with:

(1) $$P_{i}^{k}=\left(q_{pi}^{k},d_{i}^{k}\right)$$

where $q_{pi}^{k}=\left(x_{pi}^{k},y_{pi}^{k}\right)$ represents aPad GPS coordinates and $d_{i}^{k}\in N_{0}^{4}$ is the status of each aPad’s four docks. Element $d_{ij}^{k}$ in $d_{i}^{k}$ assumes a value of 0 if the dock is empty or a value in range [1,m] which corresponds to the ID number of the aMussel in dock *j*.

The state of each aMussel $M_{i}$ in step *k* can be described with:

(2) $$M_{i}^{k}=\left(q_{mi}^{k},v_{i}^{k},s_{i}^{k}\right)$$

where $q_{mi}^{k}=\left(x_{mi}^{k},y_{mi}^{k}\right)$ represents aMussel GPS coordinates, $v_{i}^{k}$ is a sensor measurement value and $s_{i}^{k}=\left(s_{i}^{k},c_{i}^{k},o_{i}^{k}\right)$ is the aMussel status vector. $s_{i}^{k}$ assumes value 1 if the aMussel is on the surface and value 0 if the aMussel is on the seabed, $c_{i}^{k}$ assumes value 1 if the aMussel is in an aPad dock and 0 otherwise, and $o_{i}^{k}$ contains information on whether the aMussel is considered to be an outlier.

During the experiments, it is assumed that aMussel coordinates $q_{mi}^{k}$ correspond to the coordinates where it was released by the aPad. The information about $s_{i}^{k}$ is determined from the position of the aMussel’s buoyancy piston, while the value of $c_{i}^{k}$ can be determined from the aMussel’s charging module which will return a value of 1 when the aMussel is connected to the aPad dock charger.

M can be presented as a union of different groups of aMussels:

(3) $$M=M_{e}\cup M_{v1}\cup \ldots \cup M_{\mathrm{vr}}$$

where $M_{e}=\left\{M_{e1},\ldots ,M_{em_{e}}\right\}$ is a group of aMussels committed to exploration and $M_{\mathrm{vi}}=\left\{M_{vi1},\ldots ,M_{vim_{vi}}\right\}$ is one of *r* groups of aMussels committed to verification. The number of elements in $M_{e}$ is $m_{e}$, and number of elements in $M_{\mathrm{vi}}$ is $m_{vi}$, where $m_{e}+m_{v1}+\ldots +m_{vr}=m$. The acoustic communication channel is such that every aMussel can hear every other aMussel in range, but they are programmed to ignore messages sent by aMussels from any group other than their own. In the experiments presented in this paper, only one verification group is used.

**Figure 4:**
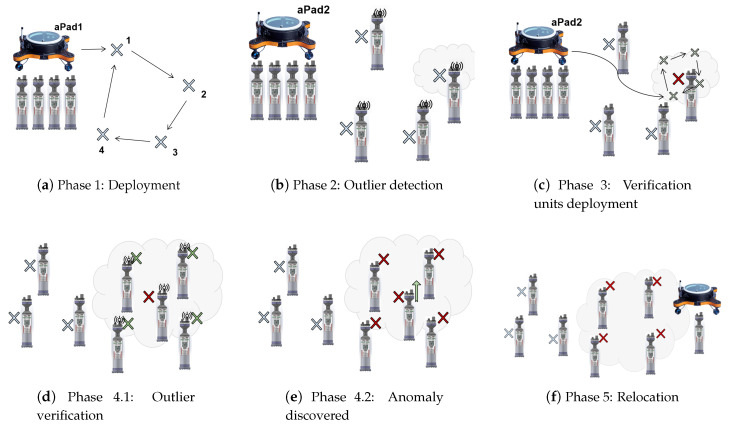
A graphical overview of the phases of the environmental monitoring scenario. (**a**) Exploration units deployment (**b**) Outlier detection (c) Verification units deployment (**d**,**e**) Outlier verification (**f**) Relocation.

As mentioned earlier, finding and confirming anomalies in a certain area of the seabed is the end goal of the proposed paradigm. Initially, all aMussels are docked to the aPads. The first step is to deploy aMussels in the exploration group $M_{e}$ over the chosen area and, in case the anomalous position is found in that area (by recognising the measurement of one aMussel as an outlier), an attempt is made to verify it by one of the verification groups of aMussels $M_{\mathrm{vl}}$. For each anomalous position found by the exploration group, one verification group is deployed. The experiment comprises several phases, which are described in the continuation and shown together in Figure 4.

#### 2.2.1. Exploration Units Deployment

The first step in the experiment includes the deployment of the $M_{e}$ group to a predefined set of GPS coordinates $q_{d}=\left\{q_{d1},\ldots ,q_{dt}\right\}$. This is achieved using the procedure described in Algorithm 1. In general, the number of predefined GPS coordinates *t* will be greater than or equal to $m_{e}$, the number of aMussels in the exploration group.
**Algorithm 1:** Deployment procedure
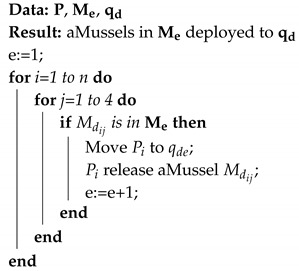


During the deployment procedure, the aPad $P_{i}$ with an exploration group aMussel $M_{d_{ij}}$ goes to the location $q_{de}$, and opens the dock containing $M_{d_{ij}}$, which releases it. This procedure is repeated for all aMussels in the exploration group.

**Figure 5:**
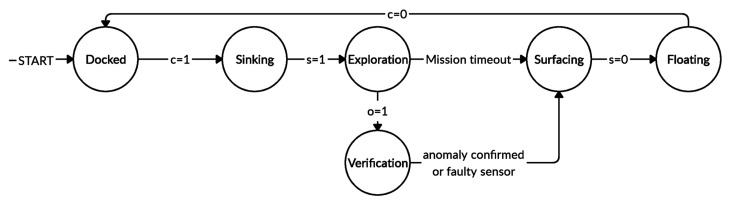
State machine of an aMussel in the exploration group.

aMussels are programmed so that on the falling edge of $c_{i}^{k}$ (which represents the moment of release from the aPad dock) they initiate the sinking procedure. The state machine of an aMussel in the exploration group is shown in Figure 5. Once again, since the aMussels start sinking immediately upon their release from the aPad dock, it is assumed that their GPS coordinates on the seabed ($q_{mi}^{k}$) correspond to the coordinates where the aPad released them ($q_{de}$) and are as such recorded by the deploying aPad. In use-cases where significant depth and strong currents are present in the target environment, acoustic localisation with aPads as surface anchors can be included in the algorithm.

**Figure 6:**
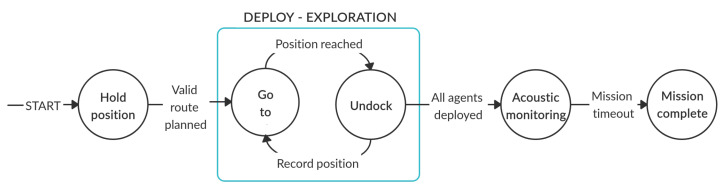
Anoxia scenario experiment for the aPad deploying exploration group agents (aPad $P_{1}$).

For the experiment presented in this paper, this step includes the aPad designated as $P_{1}$ deploying four aMussels to predefined positions, where they sink to the seabed (Figure 4a). The experimental scenario for this aPad is shown in Figure 6.

#### 2.2.2. Outlier Detection

After the aMussels from exploration group $M_{e}$ have been deployed to the designated coordinates, they start to exchange their current sensor measurement values $v_{i}^{k}$ via acoustic link, in an effort to detect any outlier in the measurements. Different algorithms for outlier detection can be used for this purpose.

For demonstration purposes, in the experiment described in this paper, a simple outlier detection algorithm was used which assumes complete communication topology between all aMussels in the group. Since all aMussels receive measurements from the rest of the group, it is possible to compute a group average. In the case when the largest individual deviation from the average value is greater than some predefined value δ, it is assumed that an outlier has been found. The choice of δ is determined heuristically, based on the expected range of the observed measurements, as well as on the value of the measurement deviation that can be considered an anomaly.

The group average in step *k* is calculated as:

(4) $$Avg^{k}=\frac{\sum_{i=1}^{m_{e}}v_{ei}^{k}}{m_{e}}$$

The maximal deviation from the average is:

(5) $$\begin{array}{c} \Delta_{max}^{k}=\max_{i\in [1,m_{e}]}|v_{ei}^{k}-Avg^{k}| \end{array}$$

where $\Delta_{max}^{k}$ is the maximal deviation from the group average in step *k*.

If the condition:

(6) $$\Delta_{max}^{k}>\delta$$

is satisfied, then the argument *o* of the outlier aMussel $M_{o}$ is:

(7) $$o=\arg\max_{i\in [1,m_{e}]}|v_{ei}^{k}-Avg^{k}|$$

The algorithm for outlier detection is presented in Algorithm 2. Data exchange between aMussels is contained in the subfuction *CalculateAverage*, where each aMussel shares its measurements ($v_{i}^{k}$) with the rest of the group and calculates the average based on the received measurements from other aMussels in the group.
**Algorithm 2:** Used outlier detection algorithm
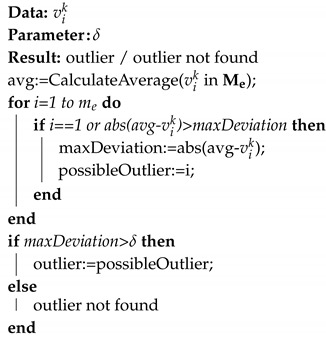


If the outlier is identified in some unit $M_{o}$, this unit adds the information that it is an outlier to its broadcast acoustic message. This broadcast designates that $M_{o}$ changes its group from $M_{e}$ to the first available verification group $M_{\mathrm{vl}}$, so the rest of the $M_{e}$ group starts ignoring messages from unit $M_{o}$. This broadcast also triggers the deployment of units that belong to the group $M_{\mathrm{vl}}$.

The implemented underwater acoustic communication is based on a time scheduling protocol using the widely-used round-robin principle presented in [31]. Time slots are assigned equally among all agents in a circular order, handling communication without priority. Each of the agents in the swarm has an equal amount of time for data sharing, which is well suited to periodically reporting a desired measured environmental variable.

The described simple outlier detection algorithm can be replaced with other outlier detection algorithms, as long as the algorithm remains distributed and suitable for operation in an underwater setting. As mentioned in the Introduction, promising approaches tested in underwater settings are, for example, median-based consensus [24] and trust-based consensus [22].

#### 2.2.3. Verification Units Deployment

Similar to during exploration units deployment, the goal of verification units deployment is for aPads to deploy a group of aMussels to the designated coordinates, where they sink to the seabed.

Once the outlier $M_{o}$ has been identified, the first available verification aMussel group $M_{\mathrm{vl}}$ is deployed to positions around the outlier $q_{mo}$ using an algorithm similar to Algorithm 1, but this time for the $M_{\mathrm{vl}}$ group. The purpose of the verification units is to verify whether the identified outlier aMussel has correct sensor readings or if it is faulty.

**Figure 7:**
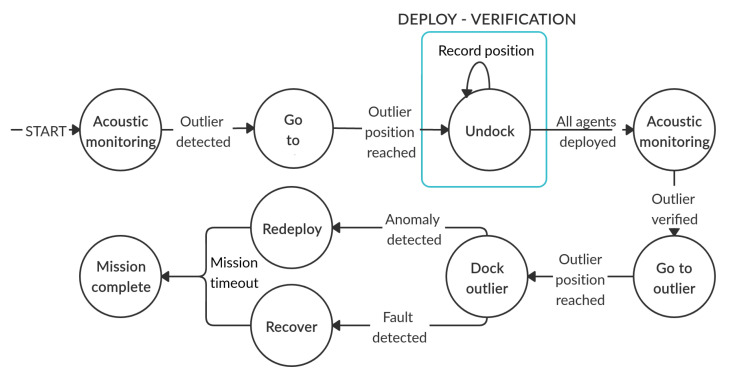
Anoxia scenario experiment for the aPad deploying verification group agents (aPad $P_{2}$).

For the experiment presented in this paper, this step includes the aPad designated as $P_{2}$ deploying four aMussels to positions around $q_{mo}$ (see Figure 4c). The experimental scenario for this aPad is shown in Figure 7.

#### 2.2.4. Outlier Verification

With the arrival of the aMussel group $M_{\mathrm{vl}}$ to the location of the outlier $M_{o}$, which is now part of the $M_{\mathrm{vl}}$ group, they start to exchange measurements in an effort to identify any potential outlier among themselves. Since all aMussels in $M_{\mathrm{vl}}$ group are located at the same coordinates, if all of them are functioning properly, their measurements should coincide. The same outlier detection algorithm as previously, presented with Equations (4)–(6), is used. In the case when no outlier is found in the group $M_{\mathrm{vl}}$, it is considered confirmed that aMussel $M_{o}$ has found the sought-after environmental anomaly, since the rest of the group $M_{\mathrm{vl}}$ has confirmed its sensor readings. In a case where $M_{o}$ is determined to be the outlier once again, it can be assumed there is something wrong with its sensors and it is presumed faulty.

#### 2.2.5. Relocation

In the event that the outlier is verified and the anomaly detection is confirmed, $M_{o}$ broadcasts a message that it is a “trusted” unit with functioning sensors. This broadcast signals to the aPads that it is ready for reallocation to the next exploration point. It also signals that $M_{o}$ is again moved from the $M_{\mathrm{vl}}$ group back to the $M_{e}$ group. After the $M_{o}$ agent surfaces, the aPad docks it autonomously and brings it to a new location for exploration (the “Redeploy” step shown in Figure 7). The rest of the $M_{\mathrm{vl}}$ group is left to monitor the anomaly.

In case of confirmed sensor fault in $M_{o}$, it again surfaces to be picked up by an aPad, but in this case it is moved to a predefined home position to await repair (the “Recover” step shown in Figure 7). In this case, the group $M_{\mathrm{vl}}$ becomes part of the exploration group $M_{e}$ and continues to monitor the environment.

In both cases, the relocation phase includes the arrival of the aPad to the coordinates of the aMussel $M_{o}$, which triggers the $M_{o}$ surfacing—when, on the surface, the aPad autonomously docks the aMussel and carries it to the selected location.

## 3. Results

The results of the proof-of-concept field experiments conducted with the swarm in order to test the viability of the proposed paradigm are presented and discussed in this section. The experiment scenario was designed around the concept of detecting and monitoring the anoxia phenomenon in the Lagoon of Venice.

### 3.1. Setup

**Figure 8:**
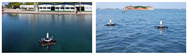
Experimental area in Biograd na Moru, Croatia (**left**); experimental area in the Venice Lagoon (**right**).

Proof-of-concept field experiments were conducted in the Adriatic Sea, in Biograd na Moru, Croatia, and Venice, Italy. The first testbed was an outdoor pool/walled off section of the sea providing a known environment for initial algorithm validation, while the second presented a “stress test” in a challenging environment on-site near Sant’Angelo della Polvere in the lagoon of Venice, Italy. In both cases, to ensure repeatability as well as to gather valuable data, the experiments took place over the span of a week, with the experimental scenario running three times a day for five days. Two examples of experiments in progress showing the swarm agents in different marine testbeds are shown in Figure 8.

**Figure 9:**
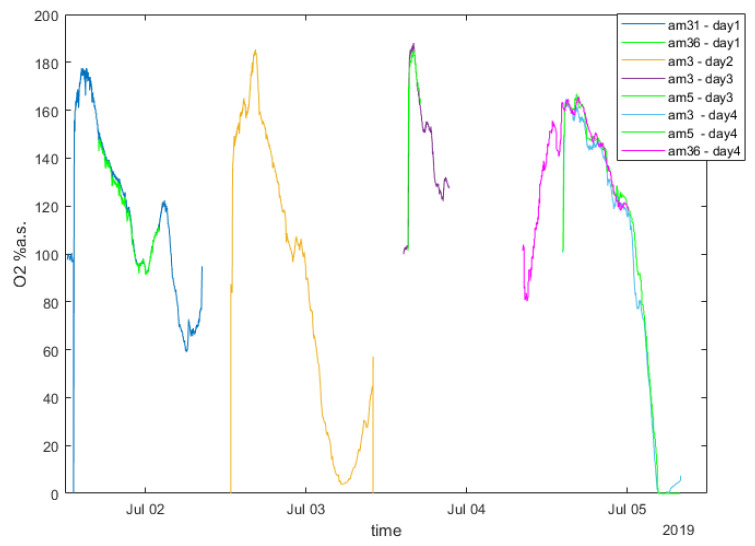
Example of oxygen data measured by aMussels.

The experiments in both testbeds were carried out by two aPad platforms and eight aMussel sensor nodes (four per aPad for full capacity, forming two groups—one for exploration and one for verification). Measurements from the aMussels’ pressure sensors were chosen as the relevant data in the proof-of-concept experiments, due to the ease of getting consistent and predictable pressure measurements of similar values in the given testbeds—in the target application of anoxia monitoring, the readings from the aMussels’ oxygen sensors would be used (Figure 9 presents the example of oxygen concentration measurements taken by aMussels over a period of four nights in the Lagoon of Venice, where a number of agents were left overnight on the seabed to monitor the oxygen levels, clearly showing nightly drops). One of the aMussels from the first group was programmed as the designated outlier, i.e., it had an offset applied to its pressure sensor readings compared to other agents in the initial exploration group. For experiments where the outlier aMussel had located an environmental anomaly, the second group of aMussels had this same offset applied to it. For experiments where the outlier aMussel had suffered some sort of fault or failure, no other aMussels had the offset applied. During both experiments, the value of δ was set to 100 hPa.

**Figure 10:**
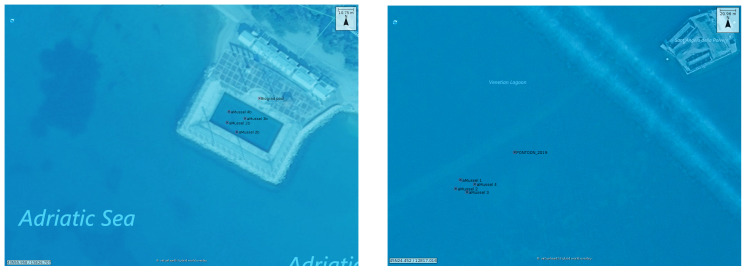
The two experimental areas: initial selected aMussel positions used in all experiments, overlaid on map with satellite image. Biograd na Moru, Croatia (**left**); Venetian Lagoon (**right**).

Four starting aMussel deployment positions were chosen within each of the experimental areas. These aMussel positions are shown in Figure 10. Repeated experiments were performed using the same starting aMussel position configurations for their respective experimental areas; however, various physical aMussel units were used interchangeably. The experiment scenarios were started on the robotic agents by human operators, and from there continued entirely autonomously.

### 3.2. Experimental Results

This section contains a detailed overview of results achieved during the proof-of-concept experiment conducted for validation purposes in an outdoor pool in Biograd na Moru, including a mission replay reconstructed from data logged on all the vehicles during the duration of the experiment. A video containing an animated showcase of this data combined with several recordings made using a drone and underwater cameras are available at http://www.youtube.com/watch?v=d4_9WKasei0.

**Figure 11:**
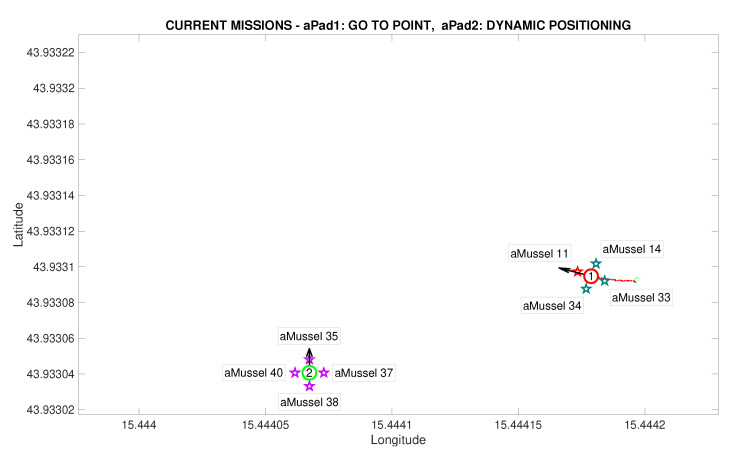
Initial deployment started in the Anoxia scenario experiment results from the deployment phase, reconstructed and replayed from recorded vehicle data—Biograd na Moru testbed.

The main points of a mission replay are shown in the following segment, illustrated by images reconstructed from real data at key points during the experiment scenario. Figure 11 depicts the initial state of the system, with two aPads holding their position, at full aMussel carrying capacity. The experiment proper then begins by aPad $P_{1}$ deploying its docked aMussel group $M_{e}$ containing aMussels 11, 14, 33, and 34 (marked in blue) to four predefined positions. The aMussel which was programmed to be the outlier in the experiment—aMussel 11—is shown in red.

**Figure 12:**
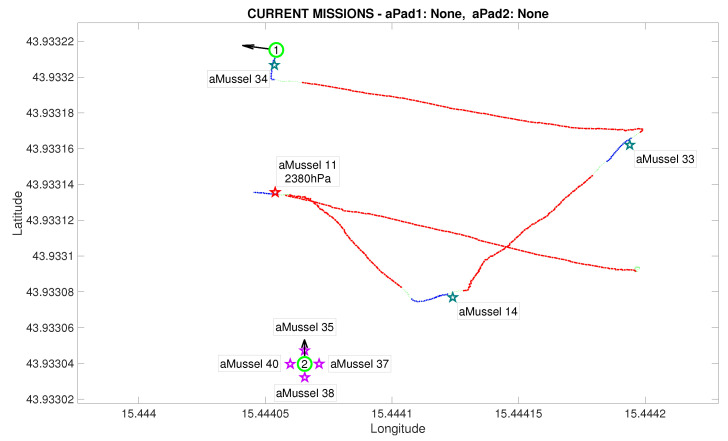
Initial deployment endeded in the Anoxia scenario experiment results from the deployment phase, reconstructed and replayed from recorded vehicle data—Biograd na Moru testbed.

**Figure 13:**
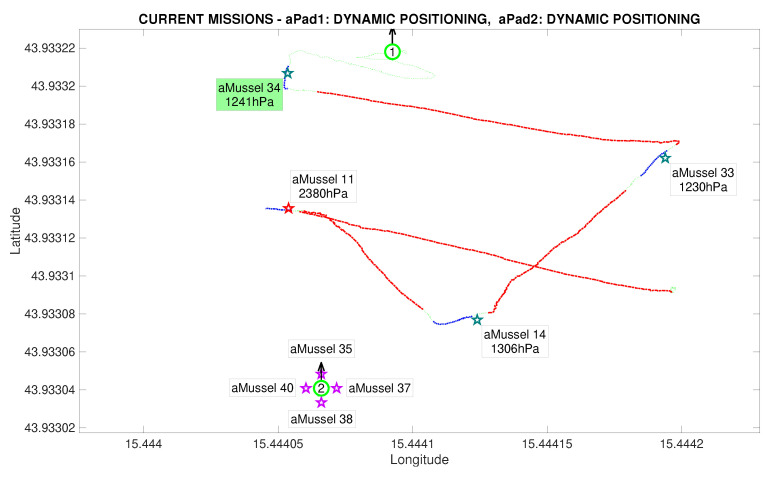
Outlier detection started in the Anoxia scenario experiment results from the deployment phase, reconstructed and replayed from recorded vehicle data—Biograd na Moru testbed.

Figure 12 shows the state after the first aMussel group has been deployed: aPad $P_{1}$ is set to hold its position near the final deployment, as all four deployed aMussels sink to the seabed where they start collecting measurements and exchanging collected information amongst themselves via acoustic communication. The aPad trajectories are plotted with colours corresponding to the mission primitive the aPad was executing at the time—go to point (red), dynamic positioning (green), or docking/undocking (blue). The labels next to each aMussel on the mission replay show the pressure data transmitted acoustically. Each aMussel broadcasts its sensor data to all other acoustic communication-capable agents in the system in a round-robin fashion, and the sending of each of these acoustic packets is indicated in the mission replay by the respective aMussel label updating and turning green, as seen in Figure 13. This marks the start of the outlier detection phase.

**Figure 14:**
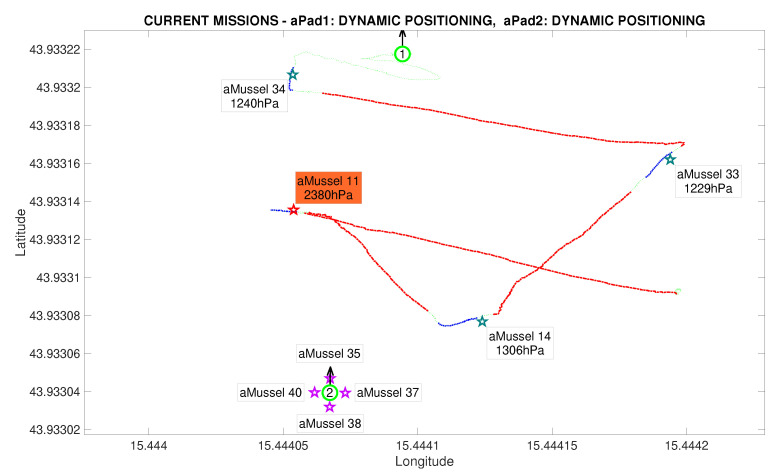
Outlier detection completed in the Anoxia scenario experiment results from the deployment phase, reconstructed and replayed from recorded vehicle data—Biograd na Moru testbed.

After exchanging a sufficient amount of data to reach a consensus, the aMussels decide if there is an outlier among them. In the event of a successful detection, the outlier aMussel (which is, as part of the exploration group, “aware” of its own outlier status) transmits the acoustic message that is the outlier to the aPads. Figure 14 shows the outlier aMussel label in orange, the agent having just reported its status, with the final pressure sensor data showing its measurements as over 1000 hPa higher than its peers.

**Figure 15:**
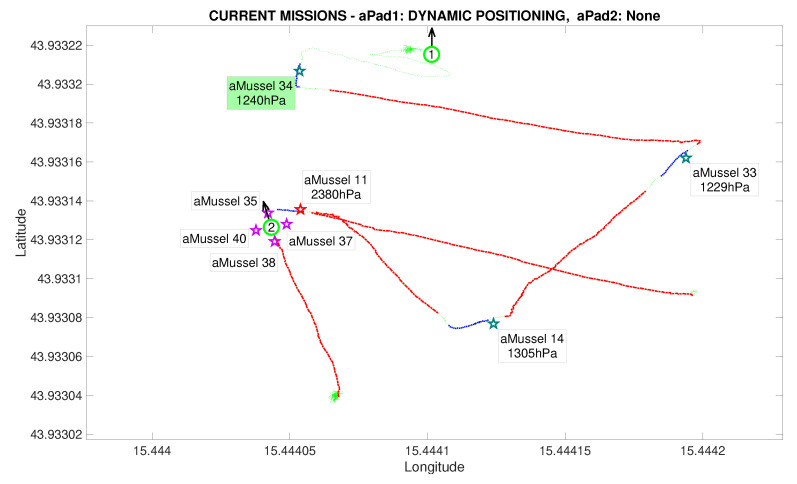
Second deployment started in the Anoxia scenario experiment results from the deployment phase, reconstructed and replayed from recorded vehicle data—Biograd na Moru testbed.

**Figure 16:**
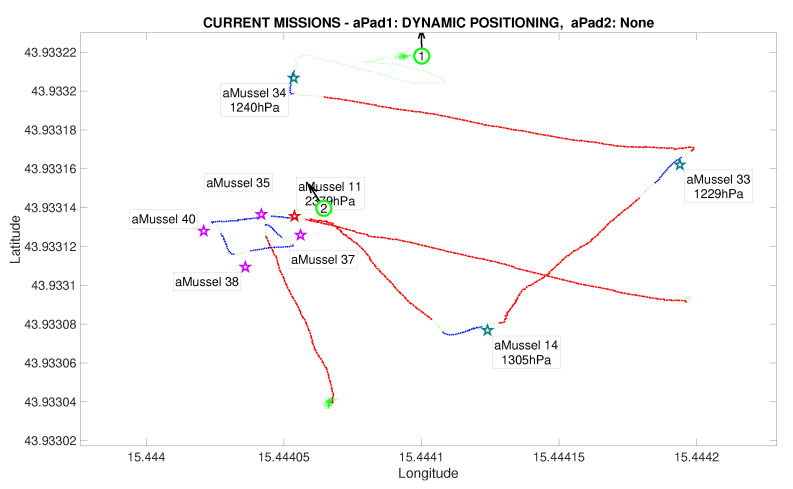
Second deployment completed in the Anoxia scenario experiment results from the deployment phase, reconstructed and replayed from recorded vehicle data—Biograd na Moru testbed.

The next phase of the experiment is verification whether the outlier aMussel is reporting on an actual environmental anomaly, or if its sensor is faulty. In this phase, aPad $P_{2}$ deploys the aMussel group $M_{\mathrm{vl}}$ containing aMussels 35, 37, 38, and 40 (marked in purple) in the vicinity of the outlier aMussel to confirm or refute its measurements, as can be seen in Figure 15 and Figure 16.

**Figure 17:**
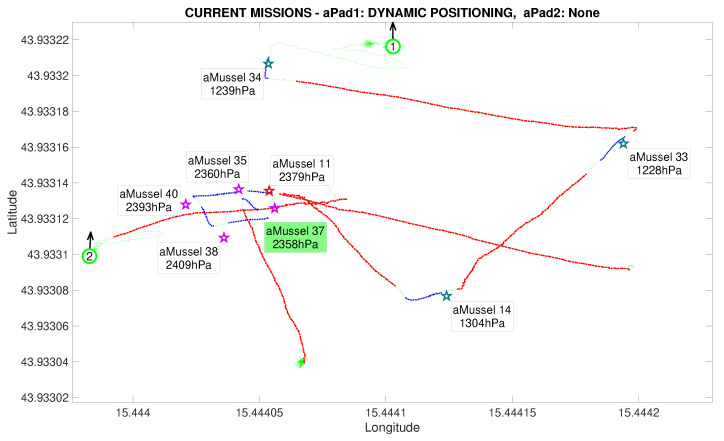
Outlier validation started in the Anoxia scenario experiment results from the deployment phase, reconstructed and replayed from recorded vehicle data—Biograd na Moru testbed.

Another instance of outlier detection is started, by having the aMussels comprising group $M_{\mathrm{vl}}$ and the original outlier exchange pressure sensor data acoustically, disregarding the data points being transmitted by the aMussels from group $M_{e}$ (Figure 17).

**Figure 18:**
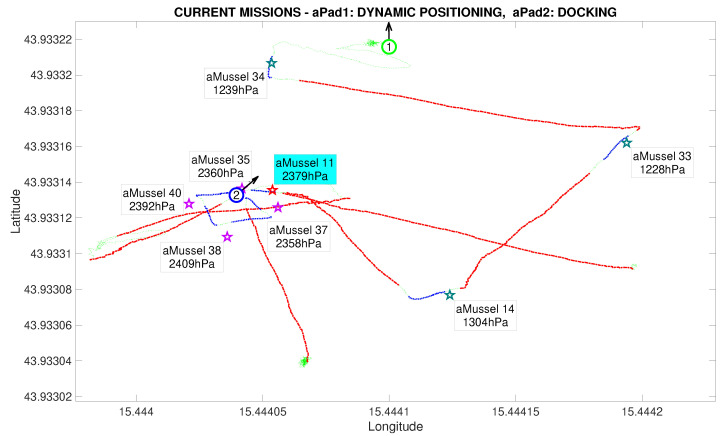
Outlier validation completed in the Anoxia scenario experiment results from the deployment phase, reconstructed and replayed from recorded vehicle data—Biograd na Moru testbed.

In this particular instance of the experiment, the assumption was that the designated outlier aMussel had indeed found an anomaly, meaning that the second group of deployed aMussels had the same programmed sensor value offset, which can be seen in their data labels. Once consensus has been reached, the now verified outlier aMussel sends a message to the aPads requesting relocation (represented by its cyan label), and the nearest available aPad moves to its position in order to collect it for further redeployment and exploration, shown in Figure 18.

**Figure 19:**
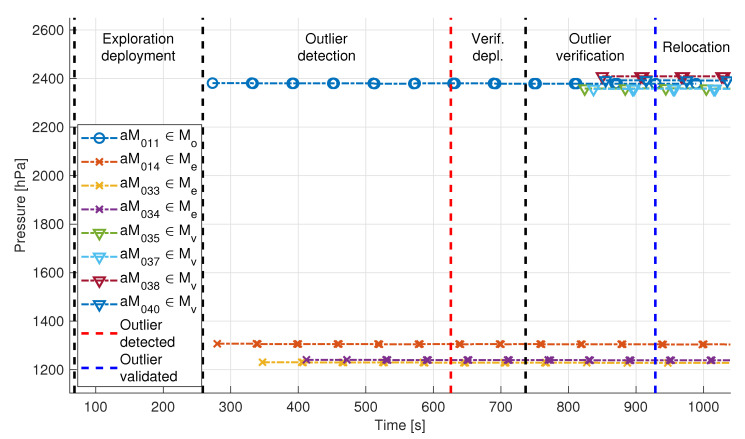
Pressure data exchanged via acoustic communication during the Biograd na Moru experiment. aMussel 11 is the outlier and was successfully verified as such.

Figure 19 shows the pressure measurements each aMussel broadcast via the acoustic channel during the Biograd na Moru experiment described above. The previously mentioned offsets of the outlier aMussel and the verification group $M_{\mathrm{vl}}$ can be clearly seen in the exchanged measurements. The data plot is divided into sections to show the system transitioning between the various phases of the algorithm.

**Figure 20:**
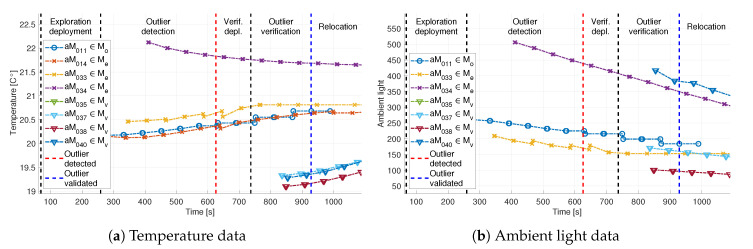
Additional sensor data exchanged via acoustic communication during the Biograd na Moru experiment.

In addition to pressure measurements, temperature and ambient light measurements were also exchanged between aMussels during the experiment, and are depicted in Figure 20 (note the consistency in data timestamps). A discrepancy in temperature data between the exploration (aM011, aM014, aM033, aM034) and verification group (aM035, aM037, aM038, aM040) is clearly apparent when looking at Figure 20a. This discrepancy is due to colder outside air temperature and the time constant of the sensor because the verification group stayed docked longer while waiting for the deployment phase to begin. However, as the agents sank to the bottom of the sea pool, the temperature started rising to the real temperature of the sea water. Additionally, aMussel 34 (purple) shows a slightly higher temperature than the other robots, possibly implying the existence of a warmer water current in the test bed.

During the “Exploration deployment” phase, there was no pressure data exchanged due to the fact that all of the aMussels were still docked on the aPads or, later, drifting on the surface and thus their acoustic modems were above water. As they sank one by one and began exchanging acoustic messages (each message is represented by a marker in the data plot), measurements started showing on the acoustic channel. The red dashed line indicates the moment that the outlier aMussel was found.

After aMussel group $M_{vl}$ was deployed, the “Outlier verification” phase began, in which the newly deployed group started to broadcast its own pressure measurements from the vicinity of the outlier aMussel. After they verified that the outlier aMussel wasn’t faulty since their pressure measurements agreed, confirming the outlier’s location as anomalous, the outlier aMussel broadcast its relocation message. This moment is indicated by a blue dashed line. The outlier’s acoustic messages stop after that point, as it surfaces for pickup (its final message was broadcast just before the 1000 s mark in the plot).

**Figure 21:**
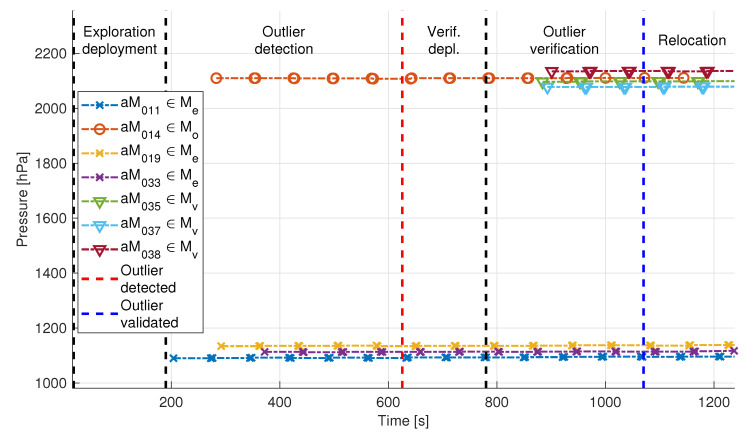
Pressure data exchanged via acoustic communication during the Venice experiment. aMussel 14 is the outlier and was successfully verified as such.

Figure 21 depicts pressure measurements exchanged during one of the instances of the experiments performed in the Lagoon of Venice. The same paradigm was tested as in the instances described above, with the designated outlier’s sensor measurements being “correct” (i.e., there was an anomaly to be discovered) and having the aMussels focusing on pressure sensor measurements. This testbed also represented a more challenging environment for the swarm—note, for example, that there is a longer delay before each of the aMussels start sending acoustic data, due to the fact that the aPads needed to cover greater distances over open water during the deployment phase and thus took longer to deploy them.

**Figure 22:**
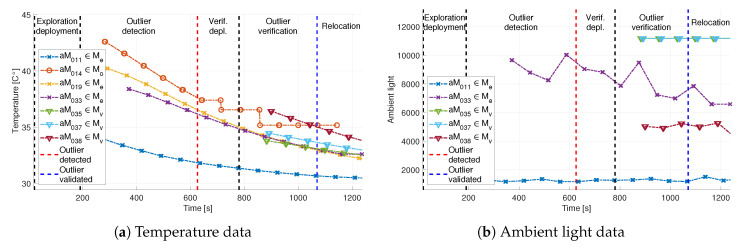
Additional sensor data exchanged via acoustic communication during the Venice experiment.

As in the Biograd na Moru experiment, Figure 22 shows the other broadcast measurements with the same consistency in data timestamps. The temperature data are displayed in Figure 22a. Before the start of the experiment, the aMussels were docked on the aPads under direct sunlight, which explains temperatures above $40{\;}^{\circ }$C. As time progresses, measured temperatures drop to normal sea temperatures in the Venice Lagoon. Ambient light data shown in Figure 22b do not contain measurements from aMussels 14 and 19. This is due to the fact that these particular aMussels were not equipped with an ambient light sensor.

The aMussel exploration group $M_{e}$ consisted of aMussels 11, 14, 19, and 33, with aMussel 14 being the designated outlier, while the verification group $M_{\mathrm{vl}}$ contained aMussels 35, 37, 38, and 39. This particular recorded dataset was chosen due to the fact that aMussel 39 from the verification group suffered a failure of its buoyancy system and did not sink to the bottom when it was supposed to, and thus did not participate in the exchange of acoustic messages for outlier verification. Nevertheless, the experiment was carried out successfully, and an environmental anomaly was determined to exist, with the outlier aMussel 14 surfacing for collection and relocation.

The step-by-step analysis of the data gathered during the proof-of-concept experiments serves to validate the functioning of the proposed distributed monitoring and fault/anomaly detection approach and demonstrate its implementation on real hardware, as well as its viability in a realistic marine environment. While the numerous functionalities and behaviours of the subCULTron swarm were tested previously (as described in [31]), this “anoxia scenario” served as a test of the swarm as a whole, relying on every aspect of it fulfilling its role, established robust communication protocols, and fault-tolerant behaviours.

The algorithm proposed and tested here makes use of the particular strengths of a distributed system in order to achieve autonomous long-term underwater monitoring, while engaging in dynamic topology reconfiguration and a type of self-maintenance by detecting and removing faulty agents from the active group. Combined with exploration strategies, the full monitoring loop could autonomously cover a significant area, creating a map of sensor values and pinpointing anomalous readings for the chosen value of interest.

## 4. Conclusions

This paper presented a novel paradigm for autonomous monitoring of a marine environment using a heterogeneous robotic swarm, as well as the field tests and experimental results achieved.

While the outlier detection algorithm used in this paper is effective in its simplicity and proved appropriate for the proof-of-concept experiments, one of the goals for continued development is testing a variety of more complex consensus algorithms, such as trust-based approaches. Another avenue of further research with the subCULTron swarm is incorporating different exploration strategies into the monitoring paradigm described here.

The main point of future work, while logistically challenging, is conducting long-term experiments while deploying the swarm at full scale. This will provide both robustness testing and gathering valuable data, as well as relevant system benchmarks helpful in identifying the main directions for potential development and system improvement. Combining the approach presented in this paper with the energy exchange and long-term autonomy related algorithms described in [32] will make it possible to use the developed swarm to its full environmental monitoring potential.

## Abbreviations

The following abbreviations are used in this manuscript:

API	Application Programming Interface
ASV	Autonomous Surface Vehicle
AUV	Autonomous Underwater Vehicle
GPS	Global Positioning System
GSM	Global System for Mobile Communications
IMU	Inertial Measurement Unit
IoUT	Internet of Underwater Things
UASN	Underwater Acoustic Sensor Network
